# Heart Rate Modeling and Prediction Using Autoregressive Models and Deep Learning

**DOI:** 10.3390/s22010034

**Published:** 2021-12-22

**Authors:** Alessio Staffini, Thomas Svensson, Ung-il Chung, Akiko Kishi Svensson

**Affiliations:** 1 Precision Health, Department of Bioengineering, Graduate School of Engineering, The University of Tokyo, Tokyo 113-8656, Japan; alessio.staffini@bocconialumni.it (A.S.); tei@bioeng.t.u-tokyo.ac.jp (U.-i.C.); akiko-kishi@umin.ac.jp (A.K.S.); 2 Department of Economics and Finance, Catholic University of Milan, 20123 Milan, Italy; 3 Business Promotion Division, ALBERT Inc., Tokyo 169-0074, Japan; 4 School of Health Innovation, Kanagawa University of Human Services Graduate School, Yokosuka 210-0821, Japan; 5 Department of Clinical Sciences, Lund University, Skåne University Hospital, 221 84 Malmo, Sweden; 6 Clinical Biotechnology, Center for Disease Biology and Integrative Medicine, Graduate School of Medicine, The University of Tokyo, Tokyo 113-0033, Japan; 7 Department of Diabetes and Metabolic Diseases, The University of Tokyo, Tokyo 113-8655, Japan

**Keywords:** heart rate, modeling, forecasting, autoregressive model, deep learning, prediction, time series analysis

## Abstract

Physiological time series are affected by many factors, making them highly nonlinear and nonstationary. As a consequence, heart rate time series are often considered difficult to predict and handle. However, heart rate behavior can indicate underlying cardiovascular and respiratory diseases as well as mood disorders. Given the importance of accurate modeling and reliable predictions of heart rate fluctuations for the prevention and control of certain diseases, it is paramount to identify models with the best performance in such tasks. The objectives of this study were to compare the results of three different forecasting models (Autoregressive Model, Long Short-Term Memory Network, and Convolutional Long Short-Term Memory Network) trained and tested on heart rate beats per minute data obtained from twelve heterogeneous participants and to identify the architecture with the best performance in terms of modeling and forecasting heart rate behavior. Heart rate beats per minute data were collected using a wearable device over a period of 10 days from twelve different participants who were heterogeneous in age, sex, medical history, and lifestyle behaviors. The goodness of the results produced by the models was measured using both the mean absolute error and the root mean square error as error metrics. Despite the three models showing similar performance, the Autoregressive Model gave the best results in all settings examined. For example, considering one of the participants, the Autoregressive Model gave a mean absolute error of 2.069 (compared to 2.173 of the Long Short-Term Memory Network and 2.138 of the Convolutional Long Short-Term Memory Network), achieving an improvement of 5.027% and 3.335%, respectively. Similar results can be observed for the other participants. The findings of the study suggest that regardless of an individual’s age, sex, and lifestyle behaviors, their heart rate largely depends on the pattern observed in the previous few minutes, suggesting that heart rate can be reasonably regarded as an autoregressive process. The findings also suggest that minute-by-minute heart rate prediction can be accurately performed using a linear model, at least in individuals without pathologies that cause heartbeat irregularities. The findings also suggest many possible applications for the Autoregressive Model, in principle in any context where minute-by-minute heart rate prediction is required (arrhythmia detection and analysis of the response to training, among others).

## 1. Introduction

Heart rate (HR) behavior is correlated with many underlying conditions and pathologies, including the risk of cardiovascular diseases [1], anxiety and depression [2,3], and asthma or breathing problems [4]. Accordingly, HR is considered an important health indicator in individuals, and forecasted HR changes may reflect changes in body state [5]. In particular, the ability to reliably and effectively predict HR fluctuations has great significance and implications for the prevention and control of some cardiovascular diseases [6].

Physiological time series are affected by many factors, making them highly nonlinear and nonstationary. As a consequence, HR time series are often considered difficult to predict and handle. Nevertheless, a number of studies have performed HR modeling. Christini et al. [7] analyzed HR dynamics and compared the performance of linear autoregressive (AR) models and linear autoregressive-moving average (ARMA) models with nonlinear models (such as the polynomial autoregressive model) and bilinear models when fitting HR data. Their results suggested that future HR dynamics should be analyzed using nonlinear methods. Saalasti [8] reviewed both linear and nonlinear classical methods for time series analysis and proposed a transistor neural network for modeling respiratory frequencies based on HR time series. Reiss et al. [9] applied convolutional neural networks to photoplethysmography (PPG)-based continuous HR and obtained promising results on large-scale datasets. Another interesting study that uses a PPG-based technique has been proposed by Gonzalez Viejo et al. [10], where a non-contact method is first proposed to accurately measure HR, and then machine learning models are developed for its prediction. Nonlinear methods have become the standard for analyzing HR time series, with principles from chaos theory, such as the Poincaré plot, being increasingly applied [11]. The above studies share high granularity of data in common, with observations often taken by the second to analyze beat-to-beat HR variability.

The last decade has seen an explosion in the availability and use of wristband-style wearable devices, notably Fitbit (Fitbit Inc., San Francisco, CA, USA) and Apple Watch (Apple Inc., Cupertino, CA, USA), and the market is expected to continue to grow [12]. The constant use of fitness trackers provides users with a variety of useful information about their HR, despite the lower accuracy and precision of these monitoring devices compared to standard electrocardiographs (ECGs) [13]. In particular, beats per minute (BPM) data can be useful for detecting possible HR anomalies on a given day, understanding how the body reacts to particular stressful or challenging situations, and accordingly providing insight on how to improve one’s own lifestyle.

The aim of the present study was to compare the performance of different forecasting models using minute-by-minute HR data and to validate the performance among multiple heterogeneous (in terms of age, sex, medical history, and smoking/drinking habits) individuals. Luo and Wu [6] previously performed a similar study; however, unlike them, this study only considered information provided by the univariate HR time series to show that the prediction results are sufficient in the absence of other physiological information on the individual, which may not be available. Other studies [14,15] considered higher frequencies (performing predictions every 30 s) but proposed a single model, validating it exclusively on a single participant and for a shorter amount of time. On the other hand, this study also compared different models with increasing complexity to draw more general conclusions on the HR patterns that were analyzed and how to best model them. Furthermore, the results of these models have been validated in multiple heterogeneous individuals. The goal of this paper is to provide the medical researchers and practitioners who often deal with HR values with a comparison between different forecasting tools and, by analyzing their results, to establish the most suitable one for minute-by-minute HR prediction. Second, this study provides a baseline on which future research could build upon in order to develop other methods that link HR with other physiological measurements. Third, by identifying a suitable architecture to capture the HR process, this study suggests that wearable devices could benefit and provide more information to their users by incorporating it.

In recent years, there has been a significant and growing application of artificial intelligence (AI) in medicine, as it has been found to be particularly useful in many clinical contexts, such as disease diagnosis [16] and precision medicine [17], and some models have managed to obtain a diagnostic accuracy comparable to that of experienced physicians. Healthcare organizations have large databases containing clinical, biological, epidemiological, and administrative data. Owing to the development of AI, the available data can be analyzed and interpreted to formulate hypotheses and obtain useful insights in developing early diagnosis, as well as targeted treatments for each patient [18]. In other words, by exploiting AI algorithms and training them on a large amount of data, it has been shown that it is possible to improve the efficacy and efficiency of current medicine.

The fields of application range from classification [19,20,21] to prediction [22,23]. Furthermore, generative adversarial networks (GANs) have been used for efficient data augmentation and to solve data privacy concerns [24,25].

The methodology and the models proposed in this study can be in principle applied to all the situations in which it is important to develop a precise forecasting model for time-series data. In the medical field, this study builds upon a fast-expanding literature that applies machine learning tools in health and healthcare forecasting. For example, the risk of the onset of a disease, whether it be cardiovascular [26,27] or not [28]; hospital discharge volume [29]; arrhythmia prevention [30], response to training in healthy individuals [31] or in individuals with pathologies [32]; identification of the existence of heart diseases [33]; or evaluation of the risk of mortality in subjects who had a heart attack during the previous year [34].

## 2. Materials and Methods

### 2.1. Study Participants

The original participants of this study were recruited from five companies in Tokyo, Japan, as part of a larger study on lifestyle interventions. Participants for the lifestyle intervention were recruited among 7437 employees who, based on the results of their completed annual health check-up, had been categorized with metabolic syndrome or at risk of metabolic syndrome. Briefly, 272 individuals were enrolled in the three-month randomized controlled trial on lifestyle changes. For the present study, the eligible individuals were only those in the intervention group (179 participants). The participants were provided with a wearable device, Fitbit Versa (Fitbit Inc., San Francisco, CA, USA; see “Wearable device” section below for details), which measured HR in BPM, among other variables, and were asked to wear it around the clock (i.e., day and night). Participants completed a mobile app-based questionnaire on lifestyle and past/present medical history.

The study was conducted in accordance with the relevant ethical guidelines and regulations in Japan. All participants received detailed information about the original study and its purpose and provided written consent to participate. The study was approved by the Ethics Committee of the School of Engineering, The University of Tokyo (approval number: KE18-44), and the research was supported by the Center of Innovation Program from the Japan Science and Technology Agency (Grant Number JPMJCE1304) and Kanagawa Prefecture’s “A project to expand the use of metabolic syndrome risk index in municipalities” (2018). The funders had no role in the study design, data collection and analysis, decision to publish, or preparation of the manuscript.

Twelve study participants with heterogeneous characteristics and HR behaviors have been selected (hereafter Participant 1, Participant 2, and Participant 3, among others) in order to evaluate the performance of the models across a diverse population. Table 1 summarizes their characteristics, including age, sex, medical history, smoking/drinking habit, and exercise habit.

Information on the number of past and present diseases, their smoking/drinking habits, and their exercise habits was extracted by a mobile app-based self-reported questionnaire administered at the start of the study.

Since the participants did not wear the device all the time, there were missing data in the recorded HR values. To obtain an evenly spaced time grid, the missing data were linearly interpolated. To minimize the cost of imputing values that were not originally recorded by the device itself, a low percentage of missing data (less than 9% across 10 days) was used as a criterion for selecting the twelve participants. For Participants 1–12, the imputed values were only 1.208%, 1.888%, 3.799%, 7.431%, 5.319%, 3.772%, 2.402%, 2.549%, 3.277%, 8.145%, 4.375%, and 2.271% of the data, respectively. The imputed values were missing at random, and there were no huge chunks of missing data.

### 2.2. Wearable Device

Fitbit Versa (FV), a consumer wearable device produced by Fitbit Inc., was used to record HR values. Details of the FV can be found on the company website (https://www.fitbit.com, accessed on 1 November 2021). Briefly, the device connects to a purpose-built smartphone app using Bluetooth technology to provide users with information about their HR (e.g., average BPM), sleep (e.g., total sleep time and efficiency), and physical activity (e.g., step count), among other variables. In normal mode, the heart rate sensor frequency is typically 5 to 15 s.

All participants in this study received instructions on how to wear FV, based on the description in the manual. Furthermore, each participant was assigned a unique username and password to enable synchronization of the device with the smartphone app throughout the study period and retrieval of the recorded data. All researchers were blinded to the participants’ username information.

### 2.3. Heart Rate

As noted above, FV provides information about users’ HR in BPM. Briefly, FV performs HR tracking based on PPG, which is an optical technique for measuring volumetric variations in the blood circulation. In the specific case of FV, green LEDs are paired with light-sensitive photodiodes, and the high-rate frequency flashing of the LEDs is used to detect volumetric changes in capillaries in the user’s wrist. Although ECG, which directly uses electrical signals produced by the heart, is generally a more accurate technique for measuring HR, recent PPG-based wearable devices have been shown to be accurate and reliable tools for HR and beat-to-beat detection [35,36].

The HR variable used in this study consisted of direct intraday observations recorded by FV. Since the goal of the present study was to model HR behavior and investigate forecasting models, slight inaccuracies in FV measurements would not constitute a huge limitation.

### 2.4. Models

To train accurate models, it is unnecessary to use observations taken across the entire study period. In particular, the analysis is restricted to data measured over a period of 10 days for a total of 14,400 observations per participant. Then, the collected data were split into a training set and a test set at a ratio of 80:20 (i.e., data from the first 8 days were used for training and those from the last 2 days were used for testing), which is reasonable given the large number of observations, and has been shown to work well in practice [37]. Individuals who led a rather constant lifestyle (although not identical) for the considered examination period were selected. If the lifestyle and the consequent HR behavior were to be very different between the training set and the test set, it would not be possible to obtain accurate forecasts without re-training the models on these new data points (since the test set would then follow a different distribution).

The performances of three different architectures with increasing complexity for minute-by-minute HR forecasting were compared. Below, the three architectures are briefly reviewed, and the motivations behind the modeling choices are explained.

All analyses were performed using Python version 3.8.3, on an Anaconda platform (Anaconda Inc., Austin, TX, USA) (release: July 2020).

#### 2.4.1. Autoregressive Process

An AR process of order p models the value of a time series at time t, $X_{t}$, using its previous p realizations $X_{t-1},\;X_{t-2},\ldots ,X_{t-p}$, according to the following formula:

$$X_{t}=\beta_{0}+\overset{p}{\underset{i=1}{\sum }}\beta_{i}X_{t-i}+\varepsilon_{t}\;,$$

where $\beta_{0}$ is the intercept of the model, $\beta_{1},\ldots \;,\beta_{p}$ are the coefficients of the lagged values, and $\varepsilon_{t}\sim WN\left(0,\sigma_{\varepsilon }^{2}\right)$. Since the realization of the variable of interest $X_{t}$ is modeled using a linear combination of the p previous realizations and a noise term, AR processes are particularly useful for modeling linear time series.

While there are multiple ways to find an adequate order p for the above process [38], analysis of the autocorrelation function (ACF) and the partial autocorrelation function (PACF) is often used. In particular, a time series following an $\mathrm{AR}\left(p\right)$ process typically shows high ACF values (even after lag p) that slowly decline and high initial PACF values that drastically decrease in magnitude after lag p.

#### 2.4.2. Long Short-Term Memory Network (LSTM)

LSTM [39] is a type of Recurrent Neural Network (RNN) that is particularly suited for sequence prediction problems, such as time series forecasting, due to the presence of memory cells that can control the flow of information. In particular, each LSTM cell contains three gates: a forget gate (which decides what information should be discarded), an input gate (which decides which values from the input should be used to update the memory state), and an output gate (which decides what to output based on the input and memory cell). The gates can be understood as weighted functions, and their parameters are updated using Backpropagation Through Time (BTT) [40]. The LSTM cell equations at a given time step t are as follows:

$$F_{t}=\sigma \left(W_{F}x_{t}+U_{F}h_{t-1}+b_{F}\right),$$

$$I_{t}=\sigma \left(W_{I}x_{t}+U_{I}h_{t-1}+b_{I}\right),$$

$${\tilde{C}}_{t}=\tanh\left(W_{C}x_{t}+U_{C}h_{t-1}+b_{C}\right),$$

$$O_{t}=\sigma \left(W_{O}x_{t}+U_{O}h_{t-1}+b_{O}\right),$$

$$C_{t}=F_{t}\circ C_{t-1}+I_{t}\circ {\tilde{C}}_{t},$$

$$h_{t}=O_{t}\circ \tanh\left(C_{t}\right),$$

where $F_{t}$, $I_{t}$, and $O_{t}$ denote the forget gate, the input gate, and the output gate, respectively; ${\tilde{C}}_{t}$ is a cell input activation vector; $C_{t}$ is the cell status; $h_{t}$ is the output vector of the LSTM cell; $x_{t}$ is the input vector to the LSTM cell; $W_{\cdot }$, $U_{\cdot }$, and $b_{\cdot }$ are the weight matrices and bias vector parameters to be tuned during the training process, respectively; σ is the sigmoid activation function; and ∘ denotes the element-wise product.

Empirical studies indicate that LSTMs often perform better in time series forecasting problems than Multi-Layer Perceptrons (MLPs) and traditional RNNs, because the presence of the gates can solve the gradient disappearance/explosion and the challenge of learning long-term dependencies [41].

To better model complexity and nonlinearity in the data, LSTM layers can be stacked on top of each other. This study used a three-layer stacked LSTM architecture. The first hidden layer contained 50 cells, the second contains 25, and the third contains 10. Dropout [42] was applied after each hidden layer to avoid overfitting: a probability of 0.3 to randomly drop each cell during training was applied. Again, following Srivastava et al. [42], dropout was combined with max-norm regularization, bounding both the weights and the biases of each hidden layer to have their norms below 3. Since this study focuses on single step forecasting, the output layer consisted of a single fully connected neuron. As the optimization algorithm, Adam [43] was used, with a learning rate of 0.001, which has been shown to work well in practice. As the activation function for the hidden layers, a hyperbolic tangent (tanh) was used, which is the standard activation function used in LSTMs, and it often allows for fast convergence [44]. As the weight initialization scheme, the Glorot uniform initializer [45], which works well for deep networks [46], was applied to each layer. The model was trained for 2000 epochs, and early stopping was employed as another form of regularization to avoid overfitting, which is often suggested in the literature [41].

Due to the high computational requirements for evaluating deep learning models, it is difficult to perform an accurate grid search for model hyperparameters. Thus, the proposed architecture was chosen following some trial and error.

#### 2.4.3. Convolutional Long Short-Term Memory Network (ConvLSTM)

ConvLSTM [47] is a variation of the LSTM described above, where convolution operations are used in the input-to-state and state-to-state transitions, reframing the problem as a spatiotemporal sequence forecasting problem. The ConvLSTM cell equations at a given time step t are as follows:

$$F_{t}=\sigma \left(W_{F}*x_{t}+U_{F}*h_{t-1}+V_{F}\circ C_{t-1}+b_{F}\right),$$

$$I_{t}=\sigma \left(W_{I}*x_{t}+U_{I}*h_{t-1}+V_{I}\circ C_{t-1}+b_{I}\right),$$

$${\tilde{C}}_{t}=\tanh\left(W_{C}*x_{t}+U_{C}*h_{t-1}+b_{C}\right),$$

$$O_{t}=\sigma \left(W_{O}*x_{t}+U_{O}*h_{t-1}+V_{O}\circ C_{t}+b_{O}\right),$$

$$C_{t}=F_{t}\circ C_{t-1}+I_{t}\circ {\tilde{C}}_{t},$$

$$h_{t}=O_{t}\circ \tanh\left(C_{t}\right),$$

where all definitions are as described for LSTM. The operator * denotes the convolution operation, and $V_{.}$ is another weight matrix used for training.

A two-layer stacked ConvLSTM architecture was developed, where the first hidden layer has 50 cells, and the second has 25. Again, since the focus of this study is single-step forecasting, the output layer consisted of a single fully connected neuron. As for the LSTM architecture, Adam was used as the optimization algorithm, tanh was used as the activation function for the hidden layers, and Glorot uniform initializer was used as the weight initialization scheme. The ConvLSTM was trained for the same number of epochs as LSTM and early stopping was applied. Again, the proposed architecture was reached after some trial and error.

## 3. Results

Before training the models, a loss function is needed. In this study, the Mean Absolute Error (MAE) was chosen, and the Root Mean Square Error (RMSE), which more greatly penalizes large errors, was used as an additional metric. Their equations are as follows:

$$\mathrm{MAE}=\frac{1}{n}\sum_{i=1}^{n}\left|{\hat{y}}_{i}-y_{i}\right|,\;\mathrm{RMSE}=\sqrt{\frac{1}{n}\sum_{i=1}^{n}{\left({\hat{y}}_{i}-y_{i}\right)}^{2}},$$

$$\mathrm{RMSE}=\sqrt{\frac{1}{n}\sum_{i=1}^{n}{\left({\hat{y}}_{i}-y_{i}\right)}^{2}},$$

where ${\hat{y}}_{i}$ and $y_{i}$ are the predicted and true HR values, respectively, over n observations.

As described in the Materials and Methods section, ACF and PACF plots were analyzed to select an appropriate order for the AR model. For all twelve participants, the plots suggested using the first 2–3 lags, the first two often being of greater importance. Table 2 summarizes the results of the $\mathrm{AR}\left(3\right)$ model on the test set.

Given that neural networks have inherent stochastic components during training (e.g., the weight initialization), their results vary at each run. Therefore, each deep learning algorithm was run 50 times for each participant.

Values indicate the mean (standard deviation) of 50 runs for each model. Text in bold denotes the best results (99% confidence level) per participant. AR(3): Autoregressive Model of order 3; LSTM: Long Short-Term Memory Network; ConvLSTM: Convolutional Long Short-Term Memory Network; MAE: Mean Absolute Error; RMSE: Root Mean Square Error.

The results in Table 2 indicate that the best performing model was the AR(3) model. While the results were closely similar between the models, an unpaired t-test confirmed that there were nevertheless statistically significantly differences in the MAE and RMSE (*p* < 0.010) for all the twelve participants. A power analysis also confirmed high statistical power (higher than 90%) for all the considered participants.

The results obtained using the training set were in line with those of the corresponding test set for each model, indicating that there was no overfitting. For completeness, the forecasts of a single run on the test set are reported in the Supplementary information for three participants (S1 File, S2 File, and S3 File).

**Figure 1:**
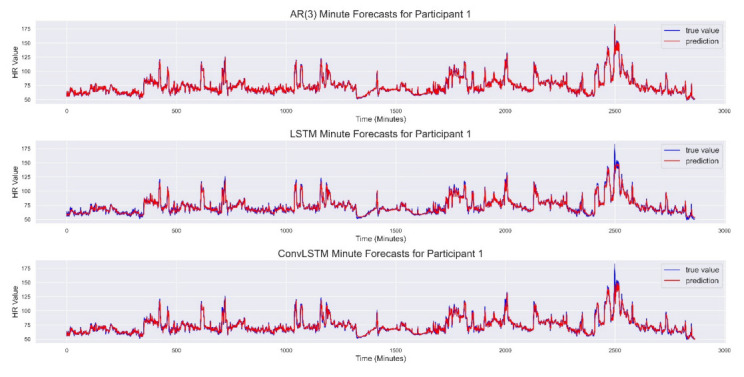
Forecast results for Participant 1. (**Top**) Results obtained from the AR(3) model. (**Center**) Results obtained from the Stacked LSTM architecture. (**Bottom**) Results obtained from the ConvLSTM architecture. AR(3): Autoregressive Model of order 3; LSTM: Long Short-Term Memory Network; ConvLSTM: Convolutional Long Short-Term Memory Network.

Figure 1 shows the minute-by-minute HR forecast generated by the three models on the test set from Participant 1.

**Figure 2:**
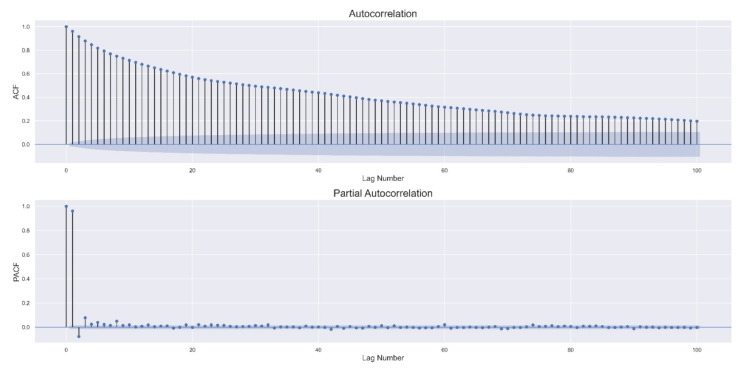
Autocorrelation function (ACF; **top**) and partial autocorrelation function (PACF; **bottom**) plots for Participant 1.

Figure 2 shows the ACF and PACF plots for Participant 1. The plots indicate the effectiveness of using an AR model.

## 4. Discussion

The goal of this study was to compare the minute-by-minute HR forecasting results of three models: a linear AR model and two deep learning models (Stacked LSTM and ConvLSTM) with an increasing order of complexity. The obtained results indicate that the three architectures performed similarly but also that the forecasts provided by the linear AR model were nevertheless statistically significantly better than the Stacked LSTM and the ConvLSTM. Furthermore, because HR fluctuations strongly depend on an individual’s innate characteristics and lifestyle behaviors, the results were validated in twelve participants heterogeneous in age, sex, and lifestyle behaviors.

The obtained results highlight three important considerations. First, the HR value at minute t is correlated with the HR values in the past three minutes and can be accurately forecasted using this information alone. Data from other participants (randomly sampled) were additionally analyzed, and the same pattern in ACF/PACF plots was observed. Second, minute-by-minute HR forecasting can be performed using data extracted from wearable devices (such as Fitbit), which collect HR information from users with relative ease, without the need for computationally demanding tools. Third, more complex models generated slightly worse HR forecasts. This is in line with the first consideration, indicating that the minute-by-minute changes in HR can be reasonably regarded as a linear process.

There are many possible extensions and applications of this work, provided that other information is available. For example, because blood oxygen saturation is often correlated with BPM [48], forecasting models for both oxygen saturation and HR fluctuations can be used to improve training plans for athletes. Alternatively, given the well-known correlation between HR and stress [49], such models can be used to measure workers’ quality of life within individual departments of a company and to optimize their daily activities according to their physiological reactions to improve their work life.

A key strength of the present study was that inter-individual differences in HR were considered when validating the models by comparing the results between heterogeneous individuals. Another strength was observing that the minute-by-minute changes in HR in individuals without rare pathologies followed a basic AR pattern. Furthermore, while the inclusion of other variables will likely improve the forecast accuracy, the considered forecasting models had relatively low error according to MAE and RMSE values.

Nevertheless, this study had several limitations that warrant consideration. First, despite using data from twelve heterogeneous participants, the forecasting models were not tested in individuals with proven pathologies that cause irregular heartbeats, such as atrial fibrillation. Second, while this study compared three different architectures and found that the AR model had the best performance, further investigation may lead to the identification of better performing models. Further, a natural extension of the work proposed here is to perform multi-step forecasting using the same three models over a longer period of time (for example, the next 10 min), and observe whether their relative performance changes. This should be examined in future research.

## 5. Conclusions

The aim of the present study was to compare three time series forecasting models for minute-by-minute HR prediction and to identify the architecture with the best performance in terms of modeling and forecasting HR behavior. Regardless of age, sex, and lifestyle, an individual’s HR in a given minute largely depends on the HR values in the previous three minutes, suggesting that minute-by-minute HR fluctuations can be reasonably considered a linear process. These findings suggest that wearable devices could implement a relatively simple model and provide useful information to their users in order to monitor anomalies in their HR values and address the risk of the onset of diseases. While other known variables related to HR fluctuations (such as the number of steps) may in principle improve the prediction, the information that HR largely depends on an individual’s HR values in the previous few minutes alone provides good forecasting results.

## Figures and Tables

**Table 1:** Characteristics of the selected participants.

	Age (Decade)	Sex	Past Diseases	Present Diseases	Smoking/Drinking Habit	Exercise Habit	Examination Period
Participant 1	30s	Female	No diseases	No diseases	Non-smoker; consumes alcohol 2–3 times per week	Exercises 1–2 days per week	10 days
Participant 2	40s	Male	3 diseases	No diseases	Past smoker; consumes alcohol 4 or more times per week	No exercise	10 days
Participant 3	50s	Male	2 diseases	1 disease	Current smoker; consumes alcohol 4 or more times per week	Exercises 1–2 days per week	10 days
Participant 4	30s	Male	No diseases	No diseases	Current smoker; consumes alcohol 2–3 times per week	No exercise	10 days
Participant 5	30s	Male	No diseases	No diseases	Non-smoker; consumes alcohol 2–4 times per month	Exercises 3 or more days per week	10 days
Participant 6	50s	Female	1 disease	1 disease	Non-smoker; consumes alcohol 4 or more times per week	Exercises 3 or more days per week	10 days
Participant 7	40s	Female	1 disease	1 disease	Non-smoker; consumes alcohol 2–4 times per month	No exercise	10 days
Participant 8	40s	Female	No diseases	No diseases	Non-smoker; consumes alcohol 2–3 times per week	No exercise	10 days
Participant 9	30s	Male	3 diseases	3 diseases	Current smoker; consumes alcohol 4 or more times per week	No exercise	10 days
Participant 10	40s	Female	No diseases	No diseases	Non-smoker; consumes alcohol 1 time or less per month	Exercises 1–2 days per week	10 days
Participant 11	50s	Male	No diseases	No diseases	Past smoker; consumes alcohol 2–4 times per month	Exercises 1–2 days per week	10 days
Participant 12	50s	Male	1 disease	1 disease	Non-smoker; consumes alcohol 4 or more times per week	Exercises 3 or more days per week	10 days

**Table 2:** Mean Absolute Error and Root Mean Square Error of the tested models.

Model	AR(3)	Stacked LSTM	ConvLSTM
Participant 1			
MAE	3.058	3.104 (0.004)	3.231 (0.011)
RMSE	4.617	4.649 (0.018)	4.984 (0.018)
Participant 2			
MAE	**2.644**	2.716 (0.039)	2.732 (0.004)
RMSE	**4.048**	4.150 (0.066)	4.271 (0.013)
Participant 3			
MAE	**2.557**	2.585 (0.021)	2.593 (0.007)
RMSE	**3.722**	3.759 (0.021)	3.792 (0.018)
Participant 4			
MAE	**3.194**	3.331 (0.005)	3.294 (0.006)
RMSE	**5.130**	5.281 (0.050)	5.453 (0.013)
Participant 5			
MAE	**2.069**	2.173 (0.024)	2.138 (0.024)
RMSE	**3.194**	3.569 (0.079)	3.420 (0.047)
Participant 6			
MAE	**2.879**	3.056 (0.036)	3.044 (0.008)
RMSE	**4.546**	5.054 (0.058)	5.026 (0.022)
Participant 7			
MAE	**2.731**	2.794 (0.022)	2.945 (0.007)
RMSE	**4.600**	4.761 (0.049)	5.038 (0.006)
Participant 8			
MAE	**2.767**	2.814 (0.051)	2.865 (0.009)
RMSE	**4.132**	4.273 (0.101)	4.425 (0.018)
Participant 9			
MAE	**2.907**	2.926 (0.008)	3.059 (0.013)
RMSE	**4.307**	4.332 (0.014)	4.471 (0.044)
Participant 10			
MAE	**2.236**	2.494 (0.082)	2.368 (0.005)
RMSE	**3.513**	4.292 (0.198)	3.857 (0.010)
Participant 11			
MAE	**2.253**	2.306 (0.012)	2.325 (0.010)
RMSE	**3.472**	3.612 (0.020)	3.693 (0.019)
Participant 12			
MAE	**3.128**	3.167 (0.010)	3.358 (0.008)
RMSE	**5.868**	6.058 (0.027)	6.527 (0.021)

## Data Availability

The authors cannot publicly provide access to individual data due to participant privacy in accordance with ethical guidelines. Additionally, the written informed consent obtained from study participants does not include a provision for publicly sharing data. Qualifying researchers may apply to access a minimal dataset upon reasonable request by contacting Saori Miyake at miyake@bioeng.t.u-tokyo.ac.jp.

## Supplementary Materials

The following are available online at https://www.mdpi.com/article/10.3390/s22010034/s1, S1 File: True and predicted HR values for Participant 1; S2 File: True and predicted HR values for Participant 2; S3 File: True and predicted HR values for Participant 3.
